# Managing health care under heavy stress: Effects of the COVID‐19 pandemic on care unit managers' ability to support the nurses—A mixed‐methods approach

**DOI:** 10.1111/jonm.13857

**Published:** 2022-10-19

**Authors:** Christian Gadolin, Maria Skyvell Nilsson, Pernilla Larsman, Anders Pousette, Marianne Törner

**Affiliations:** ^1^ School of Business, Economics and IT University West Trollhättan Sweden; ^2^ Department of Health Sciences University West Trollhättan Sweden; ^3^ Department of Psychology University of Gothenburg Gothenburg Sweden; ^4^ School of Public Health and Community Medicine, Institute of Medicine, Sahlgrenska Academy University of Gothenburg Gothenburg Sweden

**Keywords:** managers' resources, managers' role stress, perceived organizational support

## Abstract

**Aim(s):**

This study aims to investigate care unit managers' perceptions of how the COVID‐19 pandemic influenced their ability to support the nurses.

**Background:**

The COVID‐19 pandemic placed extreme pressure on health care organizations. More knowledge regarding how the pandemic influenced care unit managers' ability to support nurses is central to ensuring high‐quality health care in future crises.

**Method(s):**

A mixed‐methods study in Swedish hospitals with a survey (*n* = 128) and interviews (*n* = 20) with care unit managers.

**Results:**

Approximately half of the managers reported having spent more time available to and supporting the nurses. Availability was positively predicted by their perceived organizational support while negatively by their job demands. These job demands concerned meeting staff anxiety and managing organizational restructuring. Full focus on direct patient care and strong professional and social support were important job resources.

**Conclusion(s):**

For care unit managers to effectively support the nurses during a crisis, they need proficient job resources and moderate job demands. Managers' perceived organizational support positively affects the quality of their crisis leadership. Creating arenas in which staff collegiality can form and develop is beneficial for the ability to meet future crises.

**Implications for Nursing Management:**

This study specifies important job resources that should be acknowledged and reinforced to strengthen the ability of care unit managers to actively support the nurses during a crisis.

## 1 BACKGROUND

Effective and high‐quality health care is a cornerstone of societal welfare. Because health care work is staff‐intensive, the health and well‐being of the health care professionals is essential for the ability of health care organizations to provide high‐quality care with optimal resource utilization (Demerouti et al., 2001; Eklöf et al., 2014; Elarabi & Johari, 2014; Johnson et al., 2018). Registered nurses are fundamental for the provision of high‐quality health care at the care units and their work is highly demanding. They participate in the development of the care regimens and are vital for their implementation; they continuously monitor patients' conditions and provide essential information to physicians and other professionals in the care team; and their work is often stressful, with high cognitive and emotional demands (Chang et al., 2005; Douglas et al., 2017; Edwards & Burnard, 2003; Happell et al., 2013; Jimmieson et al., 2017). Therefore, ensuring working conditions that support nurses' ability to perform their work is essential for efficient and high‐quality health care. Previous research has identified care unit managers as central to the provision of such working conditions (Gadolin et al., 2021). As such, nurses' sound working conditions are largely dependent on their care unit manager's ability to be available at the care unit and support the nurses. Further, care unit managers themselves have emphasized that their own job demands and resources highly influence this ability, implying that the working conditions of the care unit managers also affect the working conditions of the nurses (Gadolin et al., 2022).

The COVID‐19 pandemic immensely increased the demands on health care organizations globally, affecting managers and staff at all levels (e.g., Babore et al., 2020; Eriksson et al., 2021; Sheraton et al., 2020). Nurses were at the care frontline during the COVID‐19 pandemic, (Al Thobaity & Alshammari, 2020), and their psychological burden and physical exhaustion was severe (Kishi et al., 2022). Many adverse outcomes associated with poor mental health have been identified among nurses caring for COVID‐19 patients, such as high levels of burnout (de Cordova et al., 2022), dysfunctional levels of stress and anxiety (Mo et al., 2020), as well as depression, posttraumatic stress disorder and insomnia (Varghese et al., 2021). It has been suggested that nurses who experienced high levels of organizational and social support during the COVID‐19 pandemic have been better equipped to handle the increased demands (Labrague & De los Santos, 2020). However, many nurses feel that such support is lacking (Joo & Liu, 2021). The COVID‐19 pandemic also imposed heavy demands on care unit managers who were compelled to constantly adapt to changes and manage uncertainty (Vázquez‐Calatayud et al., 2022) and balance the often contradictory needs of the nursing staff and top management (Bianchi et al., 2021), often while experiencing increased levels of stress and exhaustion (White, 2021). Given that care unit managers are central actors to ensure nurses' sound working conditions, both in general (Gadolin et al., 2021) and during the COVID‐19 pandemic (Cho et al., 2021; Qotimah et al., 2022), understanding how such pressure influences care unit managers' ability to support the nurses, and organizational stressors and resources that influence this ability, may provide health care organizations with valuable knowledge to meet future crises.

The research aim of the present study was to investigate care unit managers' perceptions of how the COVID‐19 pandemic influenced their ability to support the nurses. We did this by addressing the following research questions: (1) How did the COVID‐19 pandemic influence care unit managers' availability to support the nurses? (2) How was such availability associated with the care unit managers' own job demands and resources? (3) What specific job demands and resources did the care unit managers experience as influential for their ability to support the nurses during the pandemic?

## 2 METHODS

This mixed‐methods study comprised a questionnaire survey to all 234 care unit managers (*n* = 128, response rate 55%) at six secondary and tertiary care hospitals in two regions in western Sweden and in‐depth interviews with 20 strategically selected care unit managers at these hospitals. Mixed‐methods research has a broader focus than single method designs. The present study utilized both methods in order to provide deeper insights regarding the complex and multifaceted effects of the COVID‐19 pandemic. The quantitative data focused on establishing descriptive statistics and investigating relationships among study variables. The qualitative data aimed to openly explore the lived experiences of the care unit managers and provide more in‐depth contextualized descriptions. Therefore, the mixed‐methods design in this study may be described as being convergent (Creswell & Clark, 2017) and the methods as complementary (Greene et al., 1989), compensating for shortcomings in each respective method (cf. Giddings & Grant, 2006).

**FIGURE 1:**
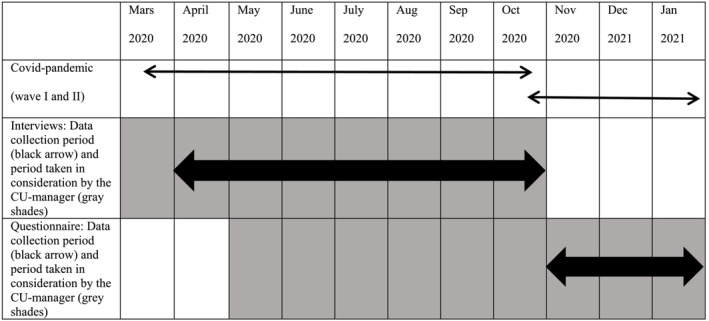
Data collection. An overview of the two data collection periods and the period considered by the care unit managers in their statements and in the questionnaire, in relation to the first two waves of the COVID‐19 pandemic in Sweden

Interviews were conducted during the first wave of the COVID‐19 pandemic in Sweden (April 2020–October 2020), while questionnaire data were collected during the second wave (November 2020–January 2021). Pen‐and‐paper questionnaires were sent out by mail to the care unit managers' workplaces and were returned directly to the researchers. In the questionnaire, the care unit managers were instructed to consider their work situation during the past 6 months when responding (Figure 1).

Starting in March 2020, the COVID‐19 virus spread rapidly in Sweden and admissions to the intensive care units were extensive (Public Health Agency of Sweden, 2022). All hospital care units were affected by the pandemic in one way or another, whether by a lack of staff, reorganization of care and staff, implementation of infection control or development of care for the COVID‐19 patients.

### 2.1 Ethical considerations

Ethics approval of the study was obtained from the Regional Ethics Committee in Gothenburg (no. 264‐18). Informed consent was provided by all participants prior to inclusion in the study. The study was conducted in accordance with the ethical standards set out in the 1964 Declaration of Helsinki and its later amendments.

### 2.2 Questionnaire

#### 2.2.1 Participants

The study sample consisted of 128 care unit managers. Eighty‐nine per cent of these were female, with a median age of 51 years (*m* = 50.9, *SD* = 7.8). Most respondents (80%) had been employed by their present regional health care organization for at least 10 years. Fifty‐nine per cent of the respondents had held the care unit manager position for more than 5 years. The number of direct subordinates per care unit manager ranged from four to 85, with a median of 34 (*m* = 32.7, *SD* = 16.1).

#### 2.2.2 Measures

##### Care unit managers' perceived job demands and resources

Three different types of care unit managers' perceived job demands were measured using items from the Gothenburg Manager Stress Inventory (GMSI) (Eklöf et al., 2010): *organizational stressors* (three items, *α* = .63; sample item ‘insufficient resources to manage peak CU loads’), *role stressors* (three items, *α* = .63; sample item ‘discord between administrative work, organizational development tasks, and subordinate contacts’) and *stressors from the subordinates* (six items, *α* = .83; sample item ‘conflicts between subordinates’). All demand items had five fixed response alternatives, ranging from ‘never/almost never’ (1) to ‘always/almost always’ (5).

The care unit managers' perceived job resources were measured using two different instruments. Two types of resources were included from the GMSI (Eklöf et al., 2010): *superior managerial support* (four items, *α* = .82; sample item ‘my superior manager shows genuine interest in what I do and the problems I face as a manager’) and *peer support* (two items, *r* = .78; sample item ‘possibilities to discuss work with colleagues’). All of these resource items had five fixed response alternatives, ranging from ‘corresponds very poorly’ (1) to ‘corresponds very well’ (5).

The care unit managers were instructed to consider their work situation during the past 6 months when responding to all of the above job demand and resource items.

The other instrument for measuring job resources was the short version (eight items, *α* = .88) of the survey of perceived organizational support (POS) (Eisenberger et al., 1986; Neves & Eisenberger, 2012). Sample items included ‘the organization really cares about my well‐being’ and ‘the organization takes pride in my accomplishments at work’. There were six fixed response alternatives, ranging from ‘completely disagree’ (1) to ‘completely agree’ (6). Four negatively worded items were reversed in the analyses.

All demand and resource variables were constructed by deriving the mean values for the items within each factor, with high values indicating high levels of demands and resources, respectively.

##### Care unit managers' perceptions of the influence of the COVID‐19 pandemic on their availability to support their staff

Care unit managers' perceptions of the influence of the COVID‐19 pandemic on their possibility to be available and supportive to their staff was assessed using a single item developed within the present study—‘Which of the following statements corresponds best with your experience? The COVID‐19 pandemic has …’—with three fixed response alternatives: ‘… caused me to spend more time being present and supportive to my subordinates’, ‘… forced me to spend less time on supporting my subordinates in their work’ and ‘… not in any tangible way influenced my work in relation to my subordinates’.

#### 2.2.3 Statistical analysis

The relationship between care unit managers' job demands and resources, and their availability to staff, was investigated by a binary logistic regression. The outcome variable was dichotomized in order to analyse why care unit managers felt that the COVID‐19 pandemic had caused them to spend more time on being available and supportive to their staff (as compared to it not influencing their work in relation to their staff, *or* forcing them to spend less time supporting their subordinates). Univariate logistic regression analyses were conducted. These analyses were based on 127 care unit managers with data for the outcome variable. All statistical analyses were conducted using SPSS version 28.0 (IBM Corp., Armonk, NY).

### 2.3 Interviews

#### 2.3.1 Participants

Twenty care unit managers from 11 medical specialties were interviewed in depth (Table 1).

**TABLE 1:** Characteristics of the participants in the interview study (*n* = 20)

Characteristics	*n* (%)	Mean (standard deviation)	Range (min/max)
Region X	11 (55%)		
Region Y	9 (45%)		
Age		53 (9.8)	40–66
Gender	Female: 18 (90%)		
	Male: 2 (10%)		
Experience as a CU manager (years)		9.4 (9.4)	2–40
Manager of care unit, medical specialty	Psychiatric acute care 1 (5%); cardiology 1 (5%); oncology 1 (5%); surgery 3 (15%); ambulance 3 (15%); geriatrics/neurology 1 (5%); psychiatric care 4 (20%); forensic psychiatry 2 (10%); internal medicine 2 (10%); acute surgery 1 (5%); urology 1 (5%)		

To ensure diversity in the organizational situation and capture diverse perspectives, the care unit managers were selected among those rating their POS as high (*n* = 7), medium (*n* = 6) or low (*n* = 7) in a previously performed questionnaire survey. The two researchers conducting the interviews (CG and MSN) were blinded to this selection. Information regarding the study and its purpose was e‐mailed to the potential participants prior to each interview. Twenty participants were considered sufficient in relation to the specificity of the aim and interview questions, participants' specific experience, interviewers' experience and knowledge and the possibility to transfer the knowledge to other contexts (cf. Malterud et al., 2016).

#### 2.3.2 Interviews and analysis

The interviews with the care unit managers were conducted as part of a comprehensive research project that investigated the specific preconditions of high POS among registered nurses in hospital care, how care unit managers act and organize their work to provide such conditions and the organizational preconditions that enable or hinder such work. During these interviews, we explored how the COVID‐19 pandemic had influenced the care unit managers' ability to work supportively. First, we asked the care unit managers to describe in as much detail as possible the three most important aspects associated with the COVID‐19 pandemic that had influenced their availability and support to the nurses. Second, we asked the care unit managers if there were any other relevant aspects associated with the COVID‐19 that had influenced their availability and support during this period.

To comply with the COVID‐19 restrictions, all interviews were performed remotely by phone or computer video conference software (Zoom). The two interviewers (CG and MSN) had extensive experience in qualitative research. Each respective interview was recorded and transcribed verbatim. The complete text material was analysed through qualitative content analysis to systematize the material into descriptive categories (Graneheim & Lundman, 2004). The analysis was inductive, with the intent to capture the lived experiences of the care unit managers supporting nurses during the COVID‐19 pandemic. All text was read by three of the authors (CG, MSN and MT) to create an overall understanding of the content. This reading revealed a consistency in the care unit managers' statements, indicating that adequate information power of the data was achieved and affirming that the sample size was sufficient (cf. Malterud et al., 2016). It became apparent during the data analysis that care unit managers' experiences of supporting nurses were deeply rooted and centred around the job demands and job resources they perceived as salient during this period. Through subsequent discussions within the research group, preliminary categories defining these job demands and job resources were identified. All statements that related to each respective job demand and resource were sorted into these categories. The final step of the qualitative analysis was to describe in depth how each specific job demand and job resource had influenced the care unit managers' ability to support the nurses. The analysis was iteratively discussed among the researchers until consensus was reached regarding categorization, content and interpretation and consistency was ensured.

## 3 RESULTS

### 3.1 The COVID‐19 pandemic's influence on care unit managers' support and availability to nurses

Fifty‐nine per cent of the care unit managers indicated that the COVID‐19 pandemic had caused them to be more present and supportive towards their staff, while 26% stated less time present and supportive. Fifteen per cent indicated that the pandemic had not influenced their work in relation to their staff.

#### 3.1.1 The relationship between job demands and stressors and care unit managers' support and availability to nurses

Table 2 presents the results from the univariate binary logistic regression analyses. These results indicated that the outcome ‘COVID‐19 pandemic leading to care unit managers being more present and supportive to their staff’ was significantly and positively associated with the care unit managers' POS (OR = 1.70, 95% CI 1.02, 2.81) and negatively associated with their organizational stressors (OR = 0.54, 95% CI 0.32, 0.91) and role stressors (OR = 0.44, 95% CI 0.26, 0.75). Thus, the chances of spending more time present and supportive were higher for care unit managers perceiving high POS and lower for those perceiving high organizational stressors and/or high role stressors. In these analyses, stressors from the subordinates, number of direct subordinates, seniority as care unit manager, superior managerial support and peer support were not significantly associated with the outcome variable.

**TABLE 2:** Results from univariate binary logistic regression analyses

	Odds ratio (95% CI)	*p*
Perceived organizational support	1.70 (1.02, 2.81)	.041
Organizational stressors	0.54 (0.32, 0.91)	.021
Role stress	0.44 (0.26, 0.75)	.002
Stressors from subordinates	0.84 (0.47, 1.51)	.558
Number of direct subordinates	0.99 (0.96, 1.01)	.235
Seniority	1.01 (0.96, 1.06)	.804
Superior managerial support	1.13 (0.74, 1.74)	.572
Peer support	1.39 (0.93, 2.08)	.113

*Note*: Odds ratios with 95% confidence intervals (CI) for univariate binary logistic regression analyses predicting the outcome variable CU managers' being more present and supportive to subordinates as a consequence of the COVID‐19 pandemic.

### 3.2 Care unit managers' perceptions of how job demands and job resources had influenced their support and availability to nurses

All of the interviews showed that the COVID‐19 pandemic had placed new and heavy demands on the care unit managers, but that it had also released work resources that enabled crisis management.

#### 3.2.1 Work demands related to restructuring and organizing work

The COVID‐19 pandemic changed the working conditions and placed new and increased demands on all managers and staff in the health care organization. It increased the demands on the care unit managers to participate in administrative meetings, which absorbed a lot of time. During the early stages of the pandemic, guidelines changed continuously, which created insecurity and frustration among the staff. At the same time, many local workplace meetings were cancelled. This deprived the workplace of a forum for discussing and solving emerging problems. The care unit managers also described that the authority to make certain types of decisions was moved from them to higher hierarchical levels in the organization, which complicated decision‐making and implementation processes for the care unit managers. Patients cancelling their appointments due to the risk of being infected at the hospital also required administration. The pandemic also introduced a new, heavy task of infection tracing at the workplace, which added to the care unit managers' workload.

The sudden and significant increase in acute care assignments induced by the COVID‐19 pandemic required staff to be moved within and between care specialties and units. New working procedures for infection protection of patients and staff must also be developed. The nurses were required to manage new patient groups, be transferred to other care units within or beyond their specific competence, work longer and more shifts, work more weekends and postpone planned leave. Also, previously planned improvements, such as increased staffing or improved work schedules, had to be postponed. The care unit managers described how this restructuring work required presence, decision‐making skills and sensitivity to the impact on the individual nurses. The care unit managers spent a lot of time preparing staff to feel role secure in the relocation to other departments, through supportive dialogue, information and training. Thoughtful consideration was also necessary to determine which of the staff could be moved to another unit and care for acutely ill COVID‐19 patients or manage intensive care.

Personally, I feel bad about having to pick out six nurse anesthetists and tell them that they had to work at the intensive care unit. It wasn't a question of it being voluntary. I had to order them to go there. It wasn't fun. One of them [a nurse] is not doing well, and we have already sent him to a crisis team. It doesn't feel good. They're not accusing me, but I really feel like I'm the one who's put him in that situation. (Participant 5, Region X)

The COVID‐19 pandemic required the care unit managers to promptly make decisions to solve acute disturbances and dilemmas in the workplace, where the information was changeable and uncertain. One manager described the situation as ‘leading in limbo’. These situations required swift decisions regarding measures to reduce the spread of infection at the unit, making changes in the staffing, isolating COVID‐19 infected patients, reorganizing patient flows due to sick staff, implementing new guidelines and disseminating information about COVID‐19 to the staff.

#### 3.2.2 Work demands related to the staff

The care unit managers described continuous and swift provision of information as a significant measure for reducing staff anxiety. The staff must be kept informed about the present situation through daily briefings. The care unit managers spent a lot of time sorting through the flow of information, compiling and disseminating the most important parts. They also described that the expectations to provide valid information as challenging: ‘The staff wants information and expects you as a manager to be able to give answers’. The less experienced care unit managers perceived this situation to be particularly challenging.

The COVID‐19 pandemic made it even more important than usual for the care unit managers to be present at the care unit and enable the staff to express and process insecurity and anxiety related to factual issues regarding the disease and implementation of guidelines and routines for the care it required. Nurses' insecurity was also related to working in new units and specialties with unfamiliar work tasks and personal anxiety regarding the risk of becoming infected. The care unit managers described an anxious climate in the workplace, where a variety of factors escalated staff concerns, such as discussions in the media, limited access to personal protective equipment and a lack of clarity regarding the use of such equipment. These needs must be addressed by acquiring facts, expressing individual concerns, sharing experiences and implementing guidelines. The care unit managers described how they spent much time each day supporting such a process by being present, listening and discussing, acknowledging feelings and following‐up on the staff's well‐being. Caring for COVID‐19 patients was also highlighted as challenging for the nurses. The care unit managers emphasized the importance of being observant and sensitive to the capacity of each individual to manage the situation.

I check in on their feelings, thoughts, and the concerns they have all the time. You have to be able to talk about feeling uneasy, and that the situation is unpleasant and difficult. You must allow yourself to acknowledge that it is a hard time. I don't think this need is special for this specific workplace, rather it is the expression of the broader human need of being able to express one's feelings and anxieties without being belittled. I think that is really important for them [the nurses]. (Participant 3, Region Y)

The care unit managers explained that the nurses needed support to solve urgent problems for which answers or solutions were not always available. These problems sometimes arose as dilemmas and therefore required discussion and reflection to be resolved in a thoughtful and supportive way. The cancellation of physical workplace meetings, due to the risk of infection among staff, affected the ability to discuss and solve such problems in the work group. To compensate for this, the care unit managers tried to be present at the unit as much as possible and communicate with the staff. They also described how they provided hands‐on support to nurses who felt insecure in their basic competence or specifically in relation to their new responsibilities. This support could entail guiding the work or taking over specific tasks.

The care unit managers also described how they tried to ‘protect’ their staff and ensure acceptable working conditions, where uncertainty would be high for a long time. They did this through such means as scheduling staff summer vacations despite the uncertainty surrounding the evolution of the pandemic that determined the possibility for vacations.

#### 3.2.3 Work resources related to work tasks or organization

The care unit managers described a health care organization that focused fully on direct patient care and prevention of the spread of the COVID‐19. They were relieved of many heavy administrative tasks and numerous development projects were paused, making the nurses who were previously engaged in these projects available to the care unit managers for direct patient care. The care unit managers experienced this concentrating on what they perceived as the core of health care, direct patient care, as highly meaningful.

The COVID‐19 pandemic required the care unit managers to spend considerable time acquiring and disseminating information to their staff, but several of the informants stated that the access to updated information from the superior management had been very good. They also experienced extensive and good support from the chief physicians and infection control experts. They reported that although the COVID‐19 pandemic disrupted previous routines and initially introduced much insecurity, the work relatively quickly found its new and functional forms.

While the COVID‐19 pandemic stopped ongoing long‐term organizational development, it also initiated organizational development. This required restructuring, which demanded time from the care unit managers for implementation and support to the nurses, but it also opened a window of opportunity for innovative thinking and development. For instance, the implementation of stringent procedures and routines for care hygiene had previously required effort and time from the care unit managers, but through the COVID‐19 pandemic, these routines were fully and swiftly implemented, saving care unit managers' time and effort. The pandemic also gave rise to new solutions that were stated to be likely to continue after the pandemic. Examples of this included reallocation of work tasks between professional groups and the introduction of short and highly frequent digital workplace meetings. The pandemic also encouraged better coordination between care units, which resulted in more flexible cooperation between units. The managers felt this was likely to be maintained after the pandemic and to have long‐term positive effects.

It [the COVID‐pandemic] has actually opened up for more flexibility and the idea that we [the care unit] are a part of the bigger organization [the hospital]. I think that has been a win. Or it will be a win in the long run. A larger organization with a common goal. This new understanding will increase the flexibility of, and cooperation within, the whole hospital. (Participant 2, Region Y)

#### 3.2.4 Work resources related to the staff

The care unit managers stated that the clear focus on patient care unified the staff around what was perceived as the core of health care work. They also described considerable social support between members of the work group through caring and looking out for each other and having informal debriefing talks.

The managers also stated that the deteriorations in the nurses' work situation regarding schedules, which involved imposing more shifts and suspended leave, called for considerable understanding and solidarity within the work group. The care unit managers appealed for such solidarity, and many testified that the staff had demonstrated a deep understanding of the situation, shown flexibility and voluntarily stepped up to take on a broad social responsibility. They also shared examples of how this had unified the work group during the COVID‐19 outbreak.

My manager came in to me, and said, ‘Now you will get an impossible assignment’. ‘Oh, shit’, I thought, ‘What is it?’ ‘You must staff the infection clinic with several nurses.’ So, I wrote a mass text message to all my nurses: ‘Now it's serious, who can help out and work this weekend?’ I staffed 27 shifts in two hours, or they [the nurses] did! I was about to start crying, it was just amazing. It may not tell you much, but it's extremely good! It is usually very difficult to solve staffing shortages. (Participant 10, Region X)

## 4 DISCUSSION

Previous research has indicated that care unit managers' presence and availability to the nurses at the care unit is an essential prerequisite for their ability to support their staff (Gadolin et al., 2021). In the present study, 59% of the care unit managers stated that the COVID‐19 pandemic had caused them to spend more time present at the care unit to support their staff. A higher presence at the care unit was more common among care unit managers who themselves perceived high POS. This indicates the importance, for the health care organization's ability to manage future crises, of providing care unit managers with robust organizational support. Moreover, because previous research has convincingly shown that POS is associated with less stress and burnout and better work performance (Kurtessis et al., 2017), managers' POS is an important resource to ensure high quality and efficiency of care, also under more normal conditions. The notion that POS is an important job resource for care unit managers to tackle the challenges ensuing the COVID‐19 pandemic has also previously gained support (Gab Allah, 2021).

Our results also showed that care unit managers who experienced high organizational stressors or high role stressors spent less time being present and available to their staff during the pandemic. These results suggest that health care organizations should aim to reduce such stressors to make the health care organization better equipped to face and manage future crises.

The results of the qualitative interviews described in more detail how the COVID‐19 pandemic influenced the managers' own job demands and resources for supporting the nurses. Other studies have highlighted similar experiences by care unit managers (e.g., Vázquez‐Calatayud et al., 2022; White, 2021). The demands related to *restructuring and organizing work* emanated from the rapidly changing environment ensuing the COVID‐19 pandemic. The care unit managers had to spend time reorganizing their own work and make swift decisions, despite uncertainty, which also influenced the organization of the nurses' work. Consequently, to provide the nurses with adequate support and handle staff anxiety, the care unit managers perceived that their demands related to *the staff* increased. These demands on the care unit managers are reflected in other research, focusing nurses' challenges and needs for managerial support during the COVID‐19 pandemic (Moradi et al., 2021).

The care unit managers stated that although the COVID‐19 pandemic initially ruptured present work structures and introduced a high degree of uncertainty, organizational structures and procedures found new functional forms fairly quickly. This was supported by resources related to *work task and organization* that were made available to the care unit managers during the pandemic, in terms of highly accessible and updated information from the superior management, and continuous support from chief physicians and infection specialists. The pandemic halted many ongoing, long‐term development projects related to the health care administration and focused all activities in the organization on managing direct patient care. This released time for the care unit manager to be more directly involved in the patient care, which benefitted their sense of meaningfulness in their work. It also facilitated their ability to be available to the nurses, which is also closely related to the direct care. These results indicate that the ability to pursue professional fulfilment, and hence be able to perform tasks perceived as close to one's ‘professional core’ (Gadolin et al., 2020), is an important resource for care unit managers not least when the organization is under heavy stress. Similar results have been highlighted regarding the nursing staff, whose ability to focus on providing patient care during the COVID‐19 pandemic allowed them to rediscover the values and meaning of being a nurse (Shin & Yoo, 2022).

The qualitative results also revealed important resources emanating from *the staff* for managing the pandemic that emerged during the crisis. The new, critical and acute situation initiated and encouraged innovative thinking and solidified good work group relations. The care unit managers testified about a high level of collegiality between the nurses, who assumed a broad social responsibility for each other and for the work performed. This finding highlights the importance of creating arenas where collegiality can form and develop, to be well equipped to meet future crises. Similarly, Ke et al. (2021) identified a high willingness among nurses to work during the pandemic and found that professional commitment and patriotism were two important factors influencing this willingness.

### 4.1 Limitations

There are certain limitations to the quantitative data. The results are based on cross‐sectional analyses, which means that conclusions about causality must be drawn with caution. Reversed and/or reciprocal effects are also possible. Another limitation is the relatively small number of respondents in the quantitative study, which makes it difficult to estimate more complex models, including interaction effects. Nonetheless, when considering both quantitative and qualitative data, the results highlight that providing care unit managers with organizational support and decreasing their organizational stressors benefit their ability to support their staff in times of crisis.

All data were collected within a Swedish context. However, despite national idiosyncrasies, we argue that the findings of this paper are also relevant outside the context of the Swedish health care system due to the similar nature of the problems (cf. Morse, 1999) that the global spread of the COVID‐19 virus caused in various national health care systems (e.g., Babore et al., 2020; Eriksson et al., 2021; Sheraton et al., 2020).

## 5 CONCLUSION

The present study underscored the centrality of care unit managers' sound working conditions for their ability to act effectively as leaders during a crisis. The study indicated that care unit managers' POS was a resource for their ability to be available and supportive to the staff during the COVID‐19 pandemic, while a high level of organizational stressors and role stress were counterproductive to this goal. High access to well‐updated information and continuous knowledge support from chief physicians and specialists on infectious diseases were other important sources of support to lead during the pandemic. Another important resource for managing the COVID‐19 crisis was a high level of collegial and social support among the nurses.

## 6 IMPLICATIONS FOR NURSING MANAGEMENT

A severe crisis, like the COVID‐19 pandemic, not only implies immense challenges for health care organizations but also reveals resources that should be acknowledged and reinforced in order to better prepare for future crises. During a severe crisis, the care unit managers need acute, active and ample support from their superior managers and medical experts in terms of continuously updated information and professional expert advice, to relieve pressure, uncertainty and unpredictability, and support swift, adjustable and innovative thinking and action. Superior management can also proactively make the organization better prepared to manage a severe crisis by sustained work to moderate organizational demands on the care unit managers and provide them with solid organizational support. The results also indicate the importance of a well‐established shared professional ideology, focusing on patient needs and the caring mission, because this was highly motivational for care unit managers and nurses alike for taking on the extreme demands required to manage and ensure a high level of care during the crisis. Providing arenas in everyday work where health care professionals' collegiality can form and develop appears as essential for the social responsibility among the professionals that was manifest during the COVID‐19 pandemic. Last but not least, the extraordinary efforts during the crisis from the care unit managers and the health care professionals should be acknowledged and the opportunity to recover, both mentally and physically, should be provided.

## CONFLICTS OF INTEREST

The authors have declared no conflict of interest.

## ETHICAL CONSIDERATIONS

Ethics approval of the study was obtained from the Regional Ethics Committee in Gothenburg (no. 264‐18). Informed consent was provided by all participants prior to inclusion in the study. The study was conducted in accordance with the ethical standards set out in the 1964 Declaration of Helsinki and its later amendments.

## Data Availability

The data that support the findings of this study are available from the corresponding author upon reasonable request.
